# Advancing the art of mosquito control: the journey of the sterile insect technique against *Aedes aegypti* in Cuba

**DOI:** 10.1186/s40249-024-01224-1

**Published:** 2024-08-29

**Authors:** René Gato, Zulema Menéndez, Misladys Rodríguez, Gladys Gutiérrez-Bugallo, María del Carmen Marquetti

**Affiliations:** 1 Sterile Insect Technique Laboratory, Department of Vector Control, Institute of Tropical Medicine Pedro Kourí, Havana, Cuba; 2 Department of Epidemiology, Institute of Tropical Medicine Pedro Kourí, Havana, Cuba; 3 Toxicology and Genetics Laboratory, Department of Vector Control, Institute of Tropical Medicine Pedro Kourí, Havana, Cuba; 4 Ecology Laboratory, Department of Vector Control, Institute of Tropical Medicine Pedro Kourí, Havana, Cuba

**Keywords:** Cuba, *Aedes aegypti*, Sterile insect technique, Vector control, Arbovirus transmission, Mosquito

## Abstract

**Background:**

*Aedes aegypti*, the primary vector of dengue, chikungunya, and Zika viruses, poses a significant public health threat worldwide. Traditional control methods using insecticides are increasingly challenged by resistance and environmental concerns. The sterile insect technique (SIT) offers an eco-friendly alternative that has been successfully applied to other insect pests. This article aims to briefly review *Ae. aegypti* management in Cuba, highlighting the accomplishments, challenges, and future directions of the SIT.

**Main body:**

Here we provide a brief summary of the extensive history of *Ae. aegypti* control efforts in Cuba. After a successful eradication campaign in the 1980s, a resurgence of dengue cases has been observed in recent years, suggesting that traditional control methods may have limited effectiveness under current conditions. In response, Cuba initiated a phased approach to develop and evaluate the feasibility of SIT for *Ae. aegypti* control, starting in 2008. Initial research focused on *Ae. aegypti* mating behavior and sterilization methods, followed by successful laboratory and semi-field trials that demonstrated population suppression. The first open-field trial in 2020 confirmed the efficacy of the SIT in reducing *Ae. aegypti* populations under real-world conditions. Currently, the research is in a phase involving a cluster-randomized superiority-controlled trial. This planned trial will compare the standard vector control program with the same program augmented by the SIT, aiming to assess the impact of the SIT on dengue incidence as the primary outcome. Implementing robust epidemiological trials to evaluate the effectiveness of the SIT is complex due to potential spillover effects from mosquito and human movement across study areas. Additionally, conducting the SIT requires significant development and operational investments. Despite these challenges, the ongoing Cuban trial holds promise for establishing the SIT as an effective and sustainable tool for *Ae. aegypti* control and for reducing the burden of mosquito-borne diseases.

**Conclusions:**

The phased evaluation conducted in Cuba confirms the efficacy of the SIT against *Ae. aegypti*, highlighting its potential for sustainable mosquito-borne disease management. The effective implementation of multi-site trials will be crucial in providing evidence of the potential of the sterile insect technique as part of a strategy to reduce the incidence of arboviral diseases.

**Graphical Abstract:**

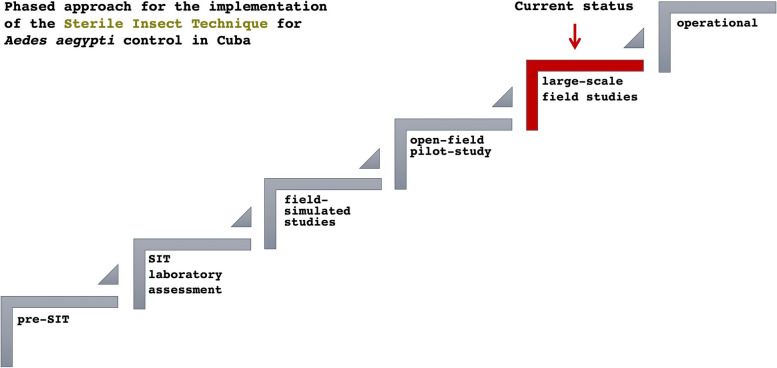

## Background

Vector-borne diseases are among the major public health concerns across the world. A safe and effective vaccine is available for yellow fever, but candidates for dengue, Zika, or chikungunya are far from ready for implementation at large scale. Therefore, the primary method of preventing epidemics worldwide is through mosquito control [1].

The major arbovirus vector in the Americas is *Aedes aegypti*, a mosquito widely distributed in urban areas throughout the tropics and subtropics. Its spread has been facilitated by the global increase in urbanization, the ever-growing human population, and the side effect of increased international travel and intercontinental trade. The potential global distribution of *Ae. aegypti* is expected to increase by 10–30% by the end of the present century as a result of climate change [1].

Nowadays, the control of mosquitoes depends to a large extent on the release of insecticides. However, it is subject to complications such as high cost, environmental impact, operational constraints, low community engagement, and inopportune timing of application. A further concern is the rapid spread of insecticide resistance, which has the potential to reduce the effectiveness of current insecticide-based control practices. Public opposition to pesticides is on the rise due to their potential impact on the environment and adverse effects on human health [2].

The drawbacks of using chemical insecticides mentioned above have led to a shift in perspective towards more sustainable methods regarding their environmental impact [2]. The sterile insect technique (SIT) is an eco-friendly method of pest control with a high potential for application against mosquito populations. The technology has been successfully applied for decades against insect pests of agricultural and veterinary importance in an area-wide integrated pest management approach [3]. Encouraged by the success of SIT in agricultural pest management, the World Health Organization (WHO) has endorsed the application of this technology as a vector control tool against *Aedes*-borne diseases [4].

The SIT is a species-specific method in which large numbers of individuals of the target species are artificially reared, irradiated to induce sexual sterility, and then released into the target population in the field. Only sterile males are released, which mate with wild-type females in the natural environment and prevent them from reproducing. If enough sterile males are released, most crosses will be sterile and the number of native insects will decrease over time. Subsequent releases will gradually increase the ratio of sterile to wild insects, resulting in a greater proportion of sterile crosses and suppression of the native population [3].

Although Cuba's vector management program was quite successful in controlling past epidemics it has not been able to achieve the same level of control in the new socioeconomic context. *Ae. aegypti* control currently relies heavily on traditional methods like insecticide spraying and larval source management. However, there is increasing interest in integrating innovative technologies, such as the SIT. This article aims to briefly review the contemporary progress of the *Ae. aegypti* management in Cuba, with emphasis on the recent achievements on SIT, their challenges and perspectives.

## Main text

### A brief history of dengue and *Ae. aegypti* surveillance and control in Cuba

The first experimental research on the transmission of febrile diseases by mosquitoes was carried out in Havana by the Cuban physician Carlos Finlay. In 1881, Finlay postulated that mosquitoes were responsible for the spread of yellow fever [5]. In 1901, the United States commission headed by Walter Reed demonstrated Finlay's theory, definitively disproving the notion of contact with fomites or human blood as the primary means of transmission. With this understanding, yellow fever was eradicated from Havana for the first time in 150 years through a remarkable program of sanitation [6].

The first report of dengue fever in the contemporary era in Cuba dates back just to 1945, but the vector control measures were not documented. No precise historical registers are available until 1953 and 1959 when the Cuban government signed an agreement with the Pan American Health Organization for vector control as part of a regional strategy [7].

A mosquito control campaign was permanently included in primary health care in 1973, which constituted the precedent of the current prevention and control programs. Between 1945 and the 1970s dengue was been unknown in Cuba, but the serotype 1 of dengue virus appeared in 1977 and produced a large-scale dengue national epidemic. More than 400,000 cases were reported, all with benign disease. There was a countrywide dispersion of *Ae. aegypti* in 1980 when the dengue virus type 2 was detected for the first time. The subsequent epidemic was recognized at the end of May 1981, after the onset of the rainy season, but had apparently begun much earlier. Dengue cases were reported simultaneously in three municipalities in the eastern, central, and western parts of Cuba. The epidemic outbreaks extended explosively to the rest of the country in a few days. A total of 344,203 cases were reported; 10,312 were classified as severe, and 158 persons died, including 101 children [7].

The Cuban Government’s response to this emergency was to launch an eradication campaign against the vector mosquito, *Ae. aegypti*. The campaign was divided into the following phases: preparatory phase (10–31 July 1981); attack phase (3 August–30 September 1981), 1-year consolidation phase (beginning 1 October 1981); and maintenance and surveillance phase (permanent). The attack phase included the elimination of mosquito breeding sites, ultra-low-volume malathion release, application of temephos as larvicide, and residual treatment with fenthion. A decree-law was approved to support sanitary inspections/treatments in houses and an intensive health education and a nationwide sanitation campaign were launched. These activities reduced the house index from 10.9% to 0.11% [8].

During the consolidation phase, all the elements of the campaign were maintained. Temporarily effective chemical spraying was progressively replaced by long-term environmental measures. A key program action was the creation of “vector controllers”, organized to find and eliminate the potential breeding sites, and to promote sanitary education. As a result of this phase, the house index was reduced from 0.11% on 30 September 1981 to 0.007% on 16 April 1984 [8].

The maintenance and surveillance phase began in January 1982 and is still in effect. Health regulations were strengthened to facilitate the operation of the vector control program. This included the right of workers to have access to all dwellings for inspections [8]. The surveillance relied on the detection -and elimination- of mosquito breeding sites. Since then, larval indices such as the house index, Breteau index, and container index have been used to estimate vector density and distribution. The deployment of traps for eggs and larvae, and the capture of adult mosquitos complemented the surveillance system [9]. The monitoring of mosquito resistance to insecticides started in 1986, following the guidelines of the WHO [10]. A comprehensive survey of insecticide resistance in Cuba is currently unavailable; however, there is local evidence indicating significant resistance to both chemical larvicides and adulticides [11].

Eradication was not achieved, but most of the 169 Cuban municipalities were free of the vector. The Cuban campaign provides an example of how *Ae. aegypti* can be successfully controlled given sufficient funds, personnel, equipment, government backing, multidisciplinary cooperation, and broad public support [8]. During the period 1982–1996, Cuba remained free of dengue. In 1997, dengue transmission reappeared in Santiago de Cuba, resulting in more than 5000 cases, 205 cases of severe dengue and 12 fatalities. Transmission was halted in approximately 6 months, mainly through sanitation, insecticide releases, and strict case isolation [7].

At the end of 2000, there were three small outbreaks of dengue in four municipalities of Havana. In June 2001, a new large epidemic began in Havana, caused by serotype 3, which extended up to 2002. In 2005, there were three small outbreaks of dengue 3 and 4 in two provinces (Havana and Camaguey). In 2006, an epidemic of serotypes 3 and 4 affected 12 of 14 provinces. The epidemic was controlled in early 2007. From 2007 to the present, dengue outbreaks have occurred continuously, with the circulation of all four viral serotypes [12].

A detailed description of the current Cuban national vector control program is beyond this article's scope. In general terms, it includes the periodic inspection of every house and institution nationwide, larval source management, biological control, education and promotion of community participation. Mosquito adulticides are reserved for outbreaks and epidemics or situations of high vector density in areas under risk [13]. Since 1986, pyrethroids (cypermethrin, lambda-cyhalothrin, deltamethrin) were approved for curative and preventive vector control, in addition to the use of organophosphates (fenthion, chlorpyrifos, malathion) and carbamates (propoxur, bendiocarb) [10].

The temephos has been applied continuously as a larvicide throughout the country for more than 30 years [14]. *Bacillus thuringiensis* was used occasionally, but there is not enough substantial proof to support its effectiveness in decreasing mosquito larval rates. There is currently no permanent mosquito monitoring system using traps, which is considered a weakness of the program. The *Aedes* indexes constitute the references for the surveillance and control activities [13].

The national program entrusts relevant studies to scientific institutions to improve the surveillance and control strategies, through a deepened understanding of the ecology and biology of *Ae. aegypti* and other mosquitoes [15, 16]. The adoption of molecular methods for virus detection in mosquitoes such as reverse transcription polymerase chain reaction (RT-PCR) has improved the studies of virus spread dynamics in nature. The vertical transmission of the dengue virus in *Ae. aegypti* has been confirmed in Cuba, hypothetically, following detection of the virus in immature stages collected in the field [17].

Over a ten-month study in Havana, larvae hatched from field-collected eggs were analyzed using RT-PCR and sequencing. Simultaneous circulation of all four dengue viruses in *Ae. aegypti* was detected, which is impressive for a relatively short space and time. The detection of dengue virus in mosquitoes may serve as an early warning sign for future outbreaks. The method is proposed for inclusion in the national program of surveillance and vector control [18].

### New approaches for vector control in Cuba

Among the spectrum of novel technologies being developed around the world for *Ae. aegypti* control [19], the SIT seemed the most feasible for Cuba at the beginning of this century. A six-phase approach was adopted for the development and evaluation of the SIT, compliant with national regulations and in line with the international framework [20].

The phases are pre-SIT (I), laboratory assessment (II), field-simulated studies (III), open-field pilot-study (IV), large-scale field studies (V), and operational (VI). Most activities are not limited in time to a particular phase, but often overlap in different phases. Some activities, such as refining each technological step or facilitating social communication, cut across all six phases.

However, a primary outcome defined for each phase is required for advancing to the next one. The primary outcome is typically implicit in the phase's designation and implies demonstrating the technology's potential for success. This is not the case for Phase I, where the prerequisite includes alignment with the national regulatory framework and obtaining consent from decision-makers to initiate the research line.

Phase I also included desk research, stakeholder engagement, fundraising, establishment of the working team, training, and capacity building. The process of gathering baseline information was initiated during this phase through the collection of entomo-epidemiological information from the health system. In subsequent phases the data collection and baseline were enhanced with the deployment of the project's own surveillance system.

The seminal research related to the SIT against *Ae. aegypti* in Cuba was initiated in 2008 as part of a technical cooperation project supported by the International Atomic Energy Agency. This project, entitled “Increasing our knowledge of male mosquito biology in relation to genetic control programmes”, grouped 21 researchers from 16 diverse countries from Africa, America, Asia and Europe [21]. The Cuban staff addressed issues related to the mating behavior of *Ae. aegypti*, which is considered critical for further progress in introducing the SIT. Additionally, the chemo-sterilization of *Ae. aegypti* was standardized.

Phase II provided a proof of concept regarding the impact of sterile males on the reproductive parameters of *Ae. aegypti* confined within laboratory cages. The chemo-sterilization did not compromise the survival or competitiveness of males. The significant decreases observed in the net reproduction rate, finite rate of natural increase, and intrinsic rate of natural increase in populations of *Ae. aegypti* treated with sterile males suggested that such populations could not proliferate in natural conditions [22].

The aim of Phase III was to assess the efficacy of releasing thiotepa-sterilized males to reduce *Ae. aegypti* populations in rooms designed to mimic natural environments. While releasing sterile males at a 2:1 ratio with fertile males yielded showed limited impact, the deployment of a 5:1 ratio proved successful in eradicating the target population in 15 weeks [23].

Between 2007 and 2014, chemicals were utilized for insect sterilization due to the absence of a viable irradiation source, posing challenges in the safe handling and disposal of alkylating agents for larger-scale studies [22, 23]. The availability of a cobalt-60 irradiator in 2014 enabled a shift towards irradiation [24], which is the preferred method worldwide [3].

Encouraged by the success of the laboratory and semi-field studies, and after a rigorous risk analysis, it was decided to launch Phase IV in 2020. For the first time in Cuba, *Ae. aegypti* mosquitoes were deliberately released. An open field trial was carried out in a suburb of Havana. The mosquito population density, and the fertility of field collected eggs were compared before and after the intervention, in both untreated control and release areas. The frequency, distance and number of mosquitoes to release were established on base of survival and dispersion of sterile males, and the wild population density, respectively, obtained by mark-release-recapture trials [25].

The Fried index reflected the high mating competitiveness of released sterile males, resulting in a marked reduction in female fecundity and a consistent decline in the field population after 12 weeks. Comparative time-series analysis of treated and control areas confirmed the success of SIT in suppressing the *Ae. aegypti* population [25]. This pilot study provided optimism for larger-scale trials to assess its impact on epidemiological outcomes.

While epidemiological field trials provide the most compelling evidence for decision-makers, designing efficacy studies for mosquito-release technologies remains a challenge. The methods advocated for establishing causal relationships in vector control effectiveness trials [4] may encounter limitations in their applicability within the area-wide approach of SIT [3] due to spillover effects resulting from mosquito migrations or human movements across study areas [4].

Despite the potential sustainability of SIT, its implementation demands significant upfront development and operational investments [3]. This highlights the importance of gathering conclusive evidence to recommend vector control interventions, as endorsed by the WHO [4].

**Fig. 1:**
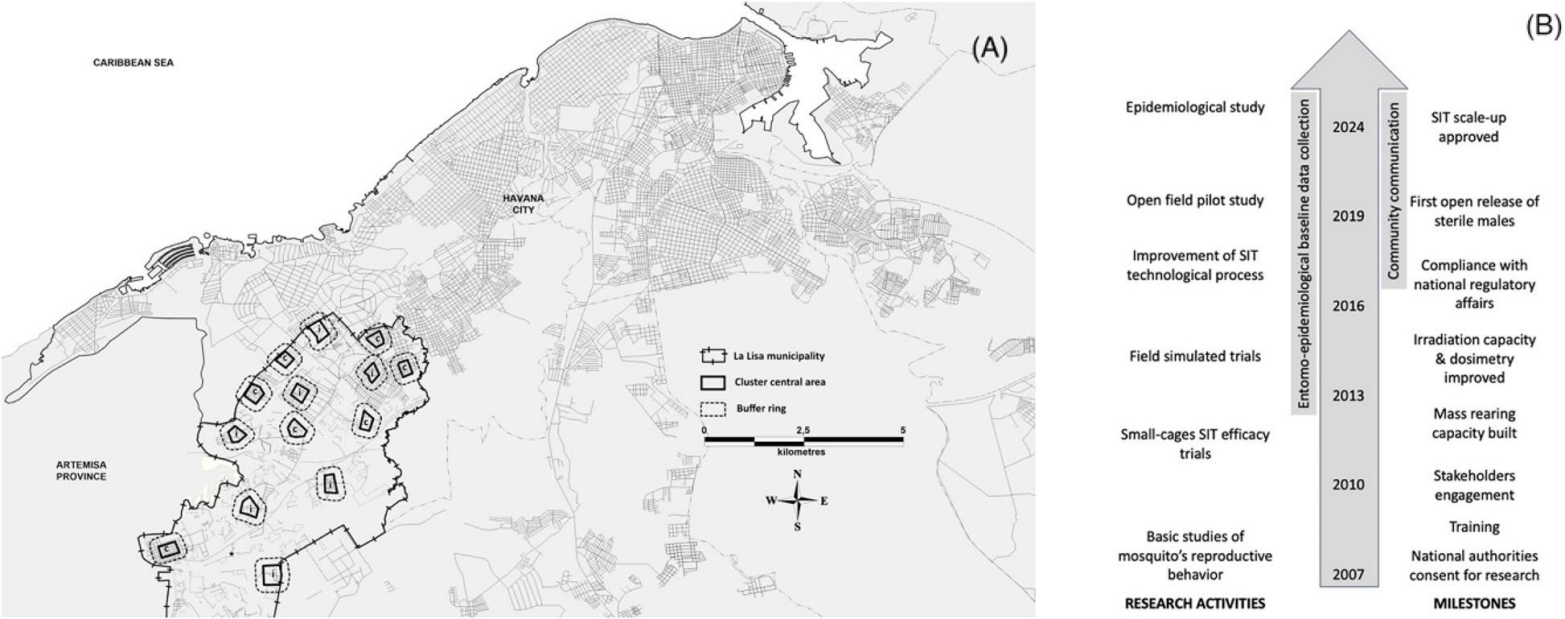
**A** Map of the western region of Havana, highlighting La Lisa municipality, the study site for evaluating the effectiveness of the SIT. The structure and location of the clusters are shown, with letters denoting the intervention (i) and control (c) clusters. **B** Timeline illustrating the historical progression of SIT development in Cuba. *SIT* sterile insect technique

The evaluation of the SIT in Cuba is currently starting its epidemiological research phase (V). A cluster-randomized superiority trial was designed according to WHO guidelines [4]. A two-arm controlled study aims to compare the efficacy of a standard vector control program (control) with the same program augmented by SIT implementation (intervention). The trial site comprises an entire municipality in Havana, divided into 14 clusters, each consisting of a 20.05–23.44-hectare central area surrounded by a 200-m buffer ring to prevent crossover effects by migration of mosquitoes (Fig. 1). The clusters were randomly assigned with a 1:1 ratio (control:intervention). The intervention will be implemented throughout the cluster, but the outcome will be measured only in the central area. The primary endpoint will be the dengue incidence, defined as the laboratory-confirmed dengue case counts in the clusters normalized by population size (rates per 1000 person-year). The cases will be confirmed as dengue according the established algorithm of the National Ministry of Health. Two entomological outcomes will be also considered primary endpoints (ovitrap index and density of eggs of *Ae. aegypti* per trap). The study plans to collect outcome data over a period of four calendar years.

## Conclusions

Cuba's long history of *Ae. aegypti* control efforts has seen notable progress with the study of innovative technologies such as the SIT. The successful implementation of SIT in laboratory and semi-field trials, followed by a promising open-field trial in 2020, underscores its potential as an effective tool in reducing *Ae. aegypti* populations. While challenges persist, including the complexity of designing epidemiological trials and the substantial initial development and operational investment required, Cuba remains committed to evaluating and advancing technologies for vector control. The ongoing epidemiological research phase, comparing standard vector control programs with SIT augmentation, reflects Cuba's openness to embracing innovative approaches such as *Wolbachia*, which offers another promising strategy in the fight against mosquito-borne diseases. Through collaborative efforts and continued research, the aim is to further contribute to the global fight against these significant public health threats.

## Data Availability

Not applicable.

## Abbreviations

SIT: Sterile insect technique
WHO: World Health Organization
RT-PCR: Reverse transcription polymerase chain reaction
